# Antiangiogenic Cancer Therapy

**DOI:** 10.1038/sj.bjc.6605032

**Published:** 2009-04-28

**Authors:** P H Maxwell, M Ashcroft

**Affiliations:** 1 Division of Medicine, Centre for Cell Signalling and Molecular Genetics, University College London, Rayne Building, 5 University Street, London WC1E 6JJ, UK

One of the most widely pursued therapeutic strategies in cancer therapy has been to target the angiogenic process in tumour progression. Angiogenesis is the process by which a solid tumour develops its own blood supply by hijacking the surrounding vasculature to invade the growing tumour mass. Angiogenesis is essential for solid tumours to grow beyond the microscopic level and metastasise. Since the seminal discoveries made on angiogenesis by Judah Folkman over three decades ago, angiogenesis has been hotly pursued as a therapeutic target in cancer. Despite this, to date, there is no effective antiangiogenic agent that can be used as a single agent in the clinic. The editors of *Antiangiogenic Cancer Therapy* have recognised the timeliness of such a book. This book provides a detailed and comprehensive overview of *Antiangiogenic Cancer Therapy*; past, present and future, making this book an important source for researchers and clinicians working in this area of cancer biology.

*Antiangiogenic Cancer Therapy* contains over 840 pages of text from 79 expert and authoritative international academic and industrial scientists and clinicians. The book is divided into four themed parts (I–IV), 1: angiogenesis and cancer, II: targeting angiogenesis for cancer therapy, III: translating angiogenesis inhibitors to the clinic and IV: treatment of specific cancers with angiogenesis inhibitors. Each part is subdivided into several chapters relevant to the theme and written by well-recognised experts on the topic of the chapter. The book spans 32 chapters. Each chapter is a comprehensive stand-alone review, including a detailed content section, an introductory discussion section, a conclusion/summary section and a comprehensive bibliography. This format allows the reader to access information in its entirety on a given topic and is aided by a very useful index at the back of the book. One minor drawback to this format is that there is some repetition, which is inevitable given the topics covered and the extensive nature of this book. The book benefits from a multitude of helpful summary tables, beautiful illustrations and drawings throughout and a section of colour figures in the centre of the book.

Part I of *Antiangiogenic Cancer Therapy* contains five chapters and provides a detailed background to angiogenesis in cancer as a basis for the proceeding parts. Chapter 1 is an interesting first chapter that discusses strategies to prolong the non-angiogenic dormant state of human cancers. Dormant tumours are pre-angiogenic where cells exhibit balanced apoptosis and proliferation. Interestingly, this chapter looks at imaging of dormant tumours and discusses molecular mechanisms and biomarkers for the angiogenic switch, metastatic dormancy and induction of dormancy using antiangiogeneic therapies. Chapter 2 follows by reviewing vascular endothelial growth factor (VEGF): basic biology and clinical implications in extensive detail. This chapter provides a background to VEGF and VEGF receptor biology, and discusses the role of VEGF signalling in physiological angiogenesis and pathological conditions, such as cancer and intraocular neovascular syndromes. The third chapter discusses angiogenesis in solid tumours and contains several informative 3-dimensional illustrations and excellent images of tumour blood vessels. Chapter 4 covers the pathophysiological role of VEGF in haematological malignancies and follows on very nicely from the earlier chapter. The final chapter of part I, chapter 5, discusses the tumour microenvironment and angiogenesis and includes an informative section on cancer metastasis.

Part II of *Antiangiogenic Cancer Therapy* introduces strategies involved in targeting angiogenesis for cancer therapy and spans 12 chapters. The second part starts with chapter 6, which provides a comprehensive review of tyrosine kinase inhibitors of angiogenesis and other multi-targeted agents, and is concluded by providing an informative perspective section. Chapter 7 deals with the development of antiangiogenic monoclonal antibodies for cancer therapy. This chapter provides a detailed background on antibody structure, discovery and engineering, describes the mechanism of action of antibody-based therapy and provides an informative section on the VEGF/VEGFR pathway and the effects of monoclonal VEGF antibody therapy (bevacizumab) in colorectal, lung, renal and breast cancer. Chapter 8 follows by focusing on FGF and the FGFR system in angiogenesis and includes both signal transduction inhibitors and dominant-negative signalling molecules. Chapter 9 covers the development of VEGF trap as a novel antiangiogenic therapy and discusses its promising preclinical evaluation in cancer models and potential use for vascular eye disease. There is some overlap in this chapter with some of the earlier chapters (e.g., VEGF biology). Interestingly, this chapter concludes by discussing the next generation targets in angiogenesis including angiopoietins and DII4. Chapter 10 follows with a detailed look at proteinases and their inhibitors in angiogenesis. A comprehensive list of metalloproteinase (MMP) inhibitor agents in the clinic (phase I–III stages) and other small molecule inhibitors (e.g., against ADAM-10) is included. This chapter also provides a transparent overview of the disappointing performance of MMP inhibitors in clinical trials. Chapter 11 focuses on prostaglandins and COX-2 and their role in antiangiogenic therapy. A useful table is included with the outcomes for various combination treatments (cytotoxic agent or a small molecule agent) with celecoxib (COX-2 inhibitor) in breast, lung, colon, pancreatic and oesophageal cancers. The role of COX-2 inhibitors in cancer treatment remains uncertain, and the chapter concludes with a helpful section on future directions for these agents. Chapter 12 discusses integrins, adhesion and coadhesion inhibitors in angiogenesis. This chapter includes a thorough background on the biology of the extracellular matrix (ECM) and the integrin family, and discusses the current status on targeting various types of integrins, for example, collagen-binding, laminin-binding, fibronectin-binding and the effects on angiogenesis. Chapter 13 provides an overview of conventional therapies with antiangiogenic activity. This chapter has some overlap with other chapters, but interestingly describes the PPAR family and inhibitors, effects of mTOR inhibitors, metronomic dosing in chemotherapy and discusses the need for combined treatment regimes when using antiangiogenic agents. Chapter 14 deals with vascular disrupting agents (VDAs) in preclinical and clinical (phase I/II) development either as single agents or in combination with radiotherapy and conventional drugs. This chapter also addresses gene therapy and ligand-based approaches to targeting the vasculature. An interesting chapter follows (chapter 15) on vascular and haematopoietic stem cells as targets for antiangiogenic therapy. Chapter 16 describes genetic strategies for targeting angiogenesis and discusses the utilisation of microarray techniques to identify downstream and upstream modulator/regulators of angiogenesis. There are several detailed sections on gene therapy-mediated delivery systems in this chapter and an excellent section on future considerations, recommendations and conclusions relating to this chapter's topic. Chapter 17 covers the identification of new targets using expression profiles with several sections including; a section on expression profiling methods, computational analysis of expression profiling data and validation of expression profiles. This chapter concludes very positively with a section on success stories in the field.

Part III of *Antiangiogenic Cancer Therapy* provides an overview of translating angiogenesis inhibitors into the clinic, including translational and clinical trial design for effective treatment using angiogenesis inhibitors. Part III starts with chapter 18, which provides background to the Federal Drug Administration (FDA) oversight of clinical trials, and includes details on the requirement for demonstration of safety and efficacy, important oncology end points, and has an informative section on the use of targeted therapies *versus* conventional cytotoxics. Chapter 19 follows, and is an important chapter, which describes surrogate markers for antiangiogeneic cancer therapy and their rationale, including the use and identification of markers in serum, plasma, urine and tumour biopsies and *ex vivo* markers. This chapter compliments the next chapter, chapter 20, which provides a detailed overview of non-invasive surrogates, and includes sections on general imaging and types of measurements, indirect measurements of vascularity and blood flow, a discussion of blood flow measurements and indirect tests not measuring blood flow (e.g., MR spectroscopy, diffusion-weighted MRI and radionuclide imaging), and details on specific (direct) imaging and current developments. This chapter has an excellent conclusions and perspectives section at the end that includes key questions regarding the role of imaging in therapy. The theme of markers continues into chapter 21, which discusses pharmacodynamic (PD) markers in tissues, including direct and indirect PD analysis of angiogenesis inhibitors and multi-targeted agents. This chapter contains several informative tables that summarise PD markers in tumours and concludes with a section on challenges and perspectives. The final chapter of part III, chapter 22 discusses blood-based biomarkers for VEGF inhibitors, outlines the importance of identifying and analysing suitable biomarkers and describes various marker types, such as soluble markers, endothelial cell markers, cellular markers in peripheral blood and markers found within other cell populations. The chapter concludes with a helpful future directions section.

Part IV of *Antiangiogenic Cancer Therapy* provides several comprehensive chapters (23–32) on the treatment of specific cancers with angiogenic inhibitors. Each chapter reviews a different cancer type, including colorectal, rectal, breast, lung and prostate cancer, haematological malignancies, glioma, Kaposi's sarcoma and melanoma. The final chapter discusses the treatment of renal cell carcinoma (RCC) with sunitinib, which is regarded as the new reference standard for the first-line treatment for RCC. It is slightly disappointing that there is no overall conclusion section at the end of the book. Several key conclusions are reiterated in the final chapters of *Antiangiogenic Cancer Therapy* that are echoed throughout the book, (a) there are limitations to antiangiogenic therapy in many cancers, and currently, most antiangiogenic agents need to be used in combination with other agents and (b) there is an increasing need for valid markers of response, improved imaging and the development of non-invasive methods for detecting response.

In summary, *Antiangiogenic Cancer Therapy* is a comprehensive book that has detailed and expertly written chapters, useful tables and beautiful illustrations. The book is excellently edited. There is absolutely no doubt that *Antiangiogenic Cancer Therapy* is an important and timely contribution to the field that is applicable to scientists and clinicians alike.

